# Correction: *Stomoxys* flies (Diptera, Muscidae) are competent vectors of *Trypanosoma evansi*, *Trypanosoma vivax*, and other livestock hemopathogens

**DOI:** 10.1371/journal.ppat.1013712

**Published:** 2025-11-25

**Authors:** Julia W. Muita, Joel L. Bargul, JohnMark O. Makwatta, Ernest M. Ngatia, Simon K. Tawich, Daniel K. Masiga, Merid N. Getahun

S1 File is uploaded incorrectly. Please see the correct S1 File here.

## Supporting information

S1 File Stomoxys Shannon diversity index, Stomoxys density, mammalian host fed on, Stomoxys feeding efficiency, primers and accession numbers. (DOCX)
