# Supplementary material for: Stomoxys flies (Diptera, Muscidae) are competent vectors of Trypanosoma evansi, Trypanosoma vivax, and other livestock hemopathogens
Source: PLoS Pathog. 2025 May 6;21(5):e1012570. doi: 10.1371/journal.ppat.1012570 (PMC12080930; doi:10.1371/journal.ppat.1012570)
Supplement: S1 File — (DOCX) [file ppat.1012570.s001.docx]

**Table A. Shannon diversity index of *Stomoxys* spp. in different counties**

| **County** | ***S. calcitrans*** | ***S. niger niger*** | ***S. boueti*** | ***S. sitiens*** | ***S. niger bilineatus*** | ***S.*** ***taeniatus*** | **Shannon Index** |
| --- | --- | --- | --- | --- | --- | --- | --- |
| Kwale | + | + | + | + | + | - | 1.36 |
| Kajiado | + | + | + | + | + | + | 1.24 |
| Kiambu | + | + | + | + | - | - | 1.14 |
| Homabay | + | + | + | + | - | - | 0.90 |
| Samburu | + | + | + | - | - | - | 0.88 |
| Marsabit | + | + | + | - | - | - | 0.83 |
| Laikipia | + | + | + | + | - | - | 0.57 |
| Isiolo | + | + | + | + | - | - | 0.47 |
| Meru | + | + | + | - | - | - | 0.29 |

**Table B. *Stomoxys* spp. count per county**

| **County** | **S*.calcitrans*** | ***S.niger niger*** | ***S.boueti*** | ***S.sitiens*** | ***S. niger bilineatus*** | ***S. taeniatus*** | **Total** |
| --- | --- | --- | --- | --- | --- | --- | --- |
| Homabay | 106 | 371 | 53 | 15 | 0 | 0 | 545 |
| Isiolo | 2204 | 215 | 34 | 61 | 0 | 0 | 2514 |
| Kajiado | 16 | 317 | 263 | 1 | 682 | 94 | 1373 |
| Kiambu | 1400 | 846 | 420 | 143 | 0 | 0 | 2809 |
| Kwale | 1207 | 1094 | 674 | 265 | 96 | 0 | 3336 |
| Laikipia | 376 | 55 | 9 | 10 | 0 | 0 | 450 |
| Marsabit | 40 | 31 | 3 | 0 | 0 | 0 | 74 |
| Meru | 183 | 7 | 6 | 0 | 0 | 0 | 196 |
| Samburu | 15 | 2 | 9 | 0 | 0 | 0 | 26 |
| **Total per spp.** | **5547** | **2938** | **1471** | **495** | **778** | **94** | **11323** |

**Table C. Table showing the count and percentage (%) of the mammalian hosts detected in blood-fed *Stomoxys***

| **Animal Host** | ***S. niger niger***  **(n=24)** | | ***S. boueti***  **(n=34)** | | ***S. calictrans***  **(n=167)** | | **Total Count** |  |
| --- | --- | --- | --- | --- | --- | --- | --- | --- |
|  | **No.** | **%** | **No.** | **%** | **No.** | **%** |  |  |
| Cattle | 11.0 | 4.9 | 13.0 | 5.8 | 65.0 | 28.9 | 89.0 |  |
| Sheep | 2.0 | 0.9 | 3.0 | 1.3 | 37.0 | 16.4 | 42.0 |  |
| Waterbuck +Buffalo | 0.0 | 0.0 | 0.0 | 0.0 | 9.0 | 4.0 | 9.0 |  |
| Warthog | 0.0 | 0.0 | 0.0 | 0.0 | 7.0 | 3.1 | 7.0 |  |
| Human | 2.0 | 0.9 | 7.0 | 3.1 | 6.0 | 2.7 | 15.0 |  |
| Buffalo | 1.0 | 0.4 | 2.0 | 0.9 | 8.0 | 3.6 | 11.0 |  |
| Giraffe | 3.0 | 1.3 | 4.0 | 1.7 | 2.0 | 0.9 | 9.0 |  |
| Goat | 0.0 | 0.0 | 0.0 | 0.0 | 8.0 | 3.6 | 8.0 |  |
| Zebra | 0.0 | 0.0 | 0.0 | 0.0 | 1.0 | 0.4 | 1.0 |  |
| Impala | 0.0 | 0.0 | 2.0 | 0.9 | 0.0 | 0.0 | 2.0 |  |
| Reedbuck | 0.0 | 0.0 | 1.0 | 0.4 | 0.0 | 0.0 | 1.0 |  |
| Elephant | 0.0 | 0.0 | 2.0 | 0.9 | 4.0 | 1.8 | 6.0 |  |
| Baboon | 1.0 | 0.4 | 0.0 | 0.0 | 0.0 | 0.0 | 1.0 |  |
| Gazelle | 4.0 | 1.8 | 0.0 | 0.0 | 0.0 | 0.0 | 4.0 |  |
| Camel | 0.0 | 0.0 | 0.0 | 0.0 | 10.0 | 4.4 | 10.0 |  |
| Cattle +goat | 0.0 | 0.0 | 0.0 | 0.0 | 2.0 | 0.9 | 2.0 |  |
| Sheep +goat | 0.0 | 0.0 | 0.0 | 0.0 | 6.0 | 2.7 | 6.0 |  |
| Camel +goat | 0.0 | 0.0 | 0.0 | 0.0 | 2.0 | 0.9 | 2.0 |  |

| Vial ID | Fly spp. | Sex | Empty vial(g) | Vial & unfed fly(g) | Fly weight(g) | Vial & fed fly(g) | Blood consumed(g) | Feeding time |
| --- | --- | --- | --- | --- | --- | --- | --- | --- |
| 1 | *S. niger niger* | F | 5.4266 | 5.4328 | 0.0062 | 5.4429 | 0.0101 | 3.45 |
| 2 | *S. niger niger* | F | 5.171 | 5.1786 | 0.0076 | 5.1968 | 0.0182 | 3.2 |
| 3 | *S. niger niger* | F | 4.9317 | 4.9403 | 0.0086 | 4.9474 | 0.0071 | 4.3 |
| 4 | *S. niger niger* | F | 5.3031 | 5.3096 | 0.0065 | 5.3239 | 0.0143 | 4.55 |
| 5 | *S. niger niger* | F | 5.4186 | 5.4232 | 0.0046 | 5.4329 | 0.0097 | 2.15 |
| 6 | *S. niger niger* | F | 5.4078 | 5.4155 | 0.0077 | 5.4187 | 0.0032 | 3.2 |
| 7 | *S. niger niger* | F | 5.6119 | 5.622 | 0.0101 | 5.6252 | 0.0032 | 6.3 |
| 8 | *S. niger niger* | F | 5.3526 | 5.3583 | 0.0057 | 5.3598 | 0.0015 | 5.5 |
| 9 | *S. niger niger* | F | 4.7919 | 4.7933 | 0.0014 | 4.8076 | 0.0143 | 5.05 |
| 10 | *S. niger niger* | F | 5.5937 | 5.617 | 0.0233 | 5.621 | 0.004 | 5.09 |
| 11 | *S. niger niger* | F | 5.4178 | 5.4265 | 0.0087 | 5.4391 | 0.0126 | 8.54 |
| 12 | *S. niger niger* | F | 5.5102 | 5.5422 | 0.032 | 5.5492 | 0.007 | 2.27 |
| 13 | *S. niger niger* | F | 5.4403 | 5.4495 | 0.0092 | 5.4693 | 0.0198 | 5.03 |
| 14 | *S. niger niger* | F | 4.8958 | 4.9028 | 0.007 | 4.9148 | 0.012 | 2.26 |
| 15 | *S. niger niger* | F | 5.8319 | 5.8378 | 0.0059 | 5.8477 | 0.0099 | 8.49 |

**Table D.** **Flies feeding efficiency on mice assay**

Note: measurement unit was gram, the consumed blood was converted to mg, which is equivalent to microliter.

**Table E**. **List of primers used for molecular identification of *Stomoxys* spp., molecular identification of livestock pathogens, and vertebrate blood meal analysis in *Stomoxys* flies**

| **Primer** | **Target gene** | **Primer sequence (5’ – 3’)** | **Product size (bp)** | **Citation** |
| --- | --- | --- | --- | --- |
| LCO1490  HCO2198 | CO1 | GGTCAACAAATCATAAAGATATTGG  TAAACTTCAGGGTGACCAAAAAATCA | 710 | [1] |
| AnaplasmaJV F  AnaplasmaJV R | *Anaplasma* 16S rRNA | CGGTGGAGCATGTGGTTTAATTC  CGRCGTTGCAACCTATTGTAGTC | 300 | [2] |
| EHR 16SD  pH1492 | *Anaplasma*/*Ehrlichia* 16S rRNA | GGTACCYACAGAAGAAGTCC  GGTTACCTTGTTACGACTT | 1030 | [3] |
| Ehrlichia16S F  Ehrlichia16S R | *Ehrlichia* 16S rRNA | CGTAAAGGGCACGTAGGTGGACTA  CACCTCAGTGTCAGTATCGAACCA | 200 | [4] |
| PER1  PER2 | *Ehrlichia* 16S rRNA | TTTATCGCTATTAGATGAGCCTATG  CTCTACACTAGGAATTCCGCTAT | 451 | [5] |
| Rick_F  Rick_R | *Rickettsia* 16S rRNA | GAACGCTATCGGTATGCTTAACACA  CATCACTCACTCGGTATTGCTGGA | 364 | [6] |
| ompB 120-2788  ompB 120-3599 | *Rickettsia ompB* | AAACAATAATCAAGGTACTGT  TACTTCCGGTTACAGCAAAGT | 836 | [7] |
| Trans1  Trans2 | *Coxiella* IS1111 | TGGTATTCTTGCCGATGAC  GATCGTAACTGCTTAATAAACCG | 687 | [8] |
| RLB F  RLB R | *Theileria*/*Babesia* 18S rRNA | GAGGTAGTGACAAGAAATAACAATA  TCTTCGATCCCCTAACTTTC | 500 | [9] |
| ITS1_CF  ITS1_BR | *Trypanosoma* sp. | CCGGAAGTTCACCGATATTG  TTGCTGCGTTCTTCAACGAA | 250 – 710 | [10] |
| Vert 16S F  Vert 16S R | Vertebrate 16S rRNA (HRM) | \| GAGAAGACCCTRTGGARCTT \| \| --- \| \| CGCTGTTATCCCTAGGGTA \| | ~200 bp | [11] |

**Appendix A. Stomoxys flies sequences deposited at the NCBI Database**

| **Fly ID** | **Accession numbers** |
| --- | --- |
| *S. niger niger* | PP587236 |
| *S. niger niger* | PP587237 |
| *S. niger niger* | PP587239 |
| *S. niger niger* | PP587238 |
| *S. boueti* | PP587243 |
| *S. taeniatus* | PQ203543 |
| *S. sitiens* | PP587235 |
| *S. sitiens* | PP587234 |
| *S. calcitrans* | PP587241 |
| *S. calcitrans* | PP587242 |
| *S. niger bilineatus* | PP587240 |
| *G. fuscipes fuscipes* | PP587247 |
| *G. fuscipes fuscipes* | PP587246 |
| *G. fuscipes fuscipes* | PP587245 |
| *G. austeni* | PP587248 |
| *G. austeni* | PP587249 |
| *G. pallidipes* | PP587244 |

**Appendix B. Pathogen sequences deposited at the NCBI Database**

| **Pathogen ID** | **Accession numbers** |
| --- | --- |
| *Th. mutans* (Ex. Mice) | PP918980 |
| *Th. mutans* (Ex. *S. calcitrans*) | PP800994 |
| *Th. mutans* (Ex. *S. calcitrans*) | PP800995 |
| *Th. taurotragi* (Ex. Cow) | PP800996 |
| *Th. velifera* (Ex. Cow) | PP800987 |
| *Th. velifera* (Ex. Cow) | PP800988 |
| *Th. velifera* (Ex. Cow) | PP800989 |
| *Th. velifera* (Ex. Cow) | PP800990 |
| *Th. orientalis* (Ex. Cow) | PP800992 |
| *Th. orientalis* (Ex. Cow) | PP800993 |
| *Th. ovis* (Ex. Cow) | PP932406 |
| *Th. ovis* (Ex. Cow) | PP800991 |
| *Babesia bigemina* (Ex. Cow) | PP800997 |
| *Eh. canis* (Ex. Cow) | PP796829 |
| *Coxiella burnetii* (Ex. Camel) | PP943171 |
| *T. congolense* (Ex. *G. pallidipes*) | PP920209 |
| *T. evansi* (Ex. Camel) | PP920210 |
| *Candidatus Anaplasma camelii* (Ex. Camel) | PP916739 |
| *Anaplasma ovis* (Ex. Cow) | PP916738 |
| *An. platys* (Ex. Cow) | PP932046 |
| *An. platys* (Ex. Cow) | PP916737 |
| *un. Anaplasma* sp. (Ex. Cow) | PP916736 |
| *un. Anaplasma* sp. (Ex. *S. calcitrans*) | PP916735 |

**REFERENCES**

1. Folmer O, Black M, Hoeh W, Lutz R, Vrijenhoek R. DNA primers for amplification of mitochondrial cytochrome c oxidase subunit I from diverse metazoan invertebrates. Mol Mar Biol Biotechnol. 1994 Oct;3(5):294–9.

2. Mwamuye MM, Kariuki E, Omondi D, Kabii J, Odongo D, Masiga D, et al. Novel *Rickettsia* and emergent tick-borne pathogens: A molecular survey of ticks and tick-borne pathogens in Shimba Hills National Reserve, Kenya. Ticks Tick-Borne Dis [Internet]. 2017 Feb 1 [cited 2024 Mar 29];8(2):208–18. Available from: https://www.sciencedirect.com/science/article/pii/S1877959X16301406

3. Parola P, Roux V, Camicas JL, Baradji I, Brouqui P, Raoult D. Detection of ehrlichiae in African ticks by polymerase chain reaction. Trans R Soc Trop Med Hyg. 2000;94(6):707–8.

4. Tokarz R, Kapoor V, Samuel JE, Bouyer DH, Briese T, Lipkin WI. Detection of Tick-Borne Pathogens by MassTag Polymerase Chain Reaction. Vector-Borne Zoonotic Dis [Internet]. 2009 Apr [cited 2025 Jan 30];9(2):147–52. Available from: https://www.liebertpub.com/doi/abs/10.1089/vbz.2008.0088

5. Goodman JL, Nelson C, Vitale B, Madigan JE, Dumler JS, Kurtti TJ, et al. Direct Cultivation of the Causative Agent of Human Granulocytic Ehrlichiosis. N Engl J Med [Internet]. 1996 Jan 25 [cited 2025 Jan 30];334(4):209–15. Available from: https://www.nejm.org/doi/full/10.1056/NEJM199601253340401

6. Nijhof AM, Bodaan C, Postigo M, Nieuwenhuijs H, Opsteegh M, Franssen L, et al. Ticks and Associated Pathogens Collected from Domestic Animals in the Netherlands. Vector-Borne Zoonotic Dis [Internet]. 2007 Dec [cited 2025 Jan 30];7(4):585–96. Available from: https://www.liebertpub.com/doi/abs/10.1089/vbz.2007.0130

7. Roux V, Raoult D. Phylogenetic analysis of members of the genus Rickettsia using the gene encoding the outer-membrane protein rOmpB (ompB). Int J Syst Evol Microbiol. 2000;50(4):1449–55.

8. Hoover TA, Vodkin MH, Williams JC. A Coxiella burnetii repeated DNA element resembling a bacterial insertion sequence. J Bacteriol. 1992;174(17):5540–8.

9. Gubbels JM, De Vos AP, Van Der Weide M, Viseras J, Schouls LM, De Vries E, et al. Simultaneous detection of bovine Theileria and Babesia species by reverse line blot hybridization. J Clin Microbiol. 1999;37(6):1782–9.

10. Njiru ZK, Constantine CC, Guya S, Crowther J, Kiragu JM, Thompson RCA, et al. The use of ITS1 rDNA PCR in detecting pathogenic African trypanosomes. Parasitol Res. 2005;95(3):186–92.

11. Omondi D, Masiga DK, Ajamma YU, Fielding BC, Njoroge L, Villinger J. Unraveling Host-Vector-Arbovirus Interactions by Two-Gene High Resolution Melting Mosquito Bloodmeal Analysis in a Kenyan Wildlife-Livestock Interface. PLOS ONE [Internet]. 2015 Jul 31 [cited 2024 Jan 6];10(7):e0134375. Available from: https://journals.plos.org/plosone/article?id=10.1371/journal.pone.0134375
