# Supplementary material for: Stomoxys flies (Diptera, Muscidae) are competent vectors of Trypanosoma evansi, Trypanosoma vivax, and other livestock hemopathogens
Source: PLoS Pathog. 2025 May 6;21(5):e1012570. doi: 10.1371/journal.ppat.1012570 (PMC12080930; doi:10.1371/journal.ppat.1012570)
Supplement: Attachment — Submitted filename: Response to Reviewers comments.docx [file ppat.1012570.s004.docx]

**Full title****: *Stomoxys* flies (Diptera, Muscidae) are competent vectors of *Trypanosoma evansi*, *Trypanosoma vivax*, and other livestock hemopathogens**

**Short title: Disease transmission dynamics by *Stomoxys* flies**

**Point-by-Point Response to Reviewers’ Comments**

Dear Editor, we are grateful for the valuable input to our work by yourself and reviewers. The important changes made include the following: revision of the formatting requirements, our aims and conclusion, and the correction of grammatical and syntax errors. Below is the list of point-by-point responses to the journal requirements and the reviewer’s comments.

**Reviewer # 1**

**Part I – Summary:**

Reviewer #1: The work describes novel and interesting in vivo experimental procedures, such as mouse transmission of parasitic pathogens. The experimental design is comprehensive and combines field work with molecular techniques and in vivo work. The statistics used is robust and methodology is well described, meriting reproducibility. Interesting insight on Stomoxys distribution feeding habits in Kenya is given. However, the work bears a significant lack of understanding of the **parasitological context**, has factual mistakes and does not bring any significant novelty.
I would emplore that the work focuses on Stomoxys distribution and behavior in Kenya (I would recommend adding the geographical location to the title of the work). The focus on pathogen transmission and host/vector competence should be generally less focused on, as this part of the work lacks novelty and is experimentally limited.

In general I would recommend a major revision of the works aims and conclusions, as these are unclear and not well delivered.

**Authors:** We would like to thank the reviewer for his/her critical constructive evaluation of our work. We have addressed your concern as the following

With regards to the title, we believe that limiting the title to a specific location may reduce its broader relevance as stomoxys are cosmopolitans. Our findings contribute to the general understanding of Stomoxys and their vector competence, which extends beyond Kenya and we strongly believe it applies to other regions where these flies are present. Therefore, we respectfully chosen to keep the title without a geographical reference, but we modified by listing some of the pathogens as requested.

In our work parasitology is the major component as we characterized various parasites both in livestock and vectors followed by transmission experiment in vivo. While we acknowledge that some aspects may not be entirely novel, but our findings reinforce various claims and speculations about Stomoxys as vector of various pathogens, that was not supported with limited data with strong data. Furthermore, we showed trypanosomes transmission to healthy mice by Stomoxys within one minute time span demonstrating how efficient they are. We clearly demonstrate the wide distribution, polyphagous feeding habit of stomoxys on diverse wild and domestic animals and finally showed the significance of Stomoxys as vectors of multiple hemopathogens they harbour in vivo, providing useful information about the role of stomoxys and open new areas for future research and disease control efforts and policy makers. We have addressed each of your point as follows

**Reviewer # 1**

**Part II – Major Issues: Key Experiments Required for Acceptance**

Line 151-164 describe PCR detection of hemopathogens. Did negative controls include uninfected samples to assess the viability of primers, or was only water used? Were positive controls used as well for the selected pathogens? How well do the primers distinguish between different and related species (such as T. evansi, T. vivax and T. brucei)? This should be well assessed and described in the work in order to draw further conclusions from the experimental data.

**Authors:** For negative control, nuclease-free water was used as a no-template control. This was to check for contamination and non-specific amplification. For each PCR run, we used previously confirmed DNA samples as positive controls for the selected pathogens to verify successful amplification and conditions used. This has been added to the methodology. The primers used in this study were selected based on known specificity for their respective target genes. For pathogen detection, *Anaplasma, Ehrlichia, Rickettsia, Coxiella, and Theileria/ Babesia* primers targeted conserved regions. Secondary amplification using longer primers was performed in cases where further resolving was needed. We used the ITS1 primer, which targets the conserved ITS1 region, which distinguishes *Trypanosoma* spp. based on amplicon size. As it is difficult to differentiate based on ITS-1 *T. evansi* and *T. brucei*, we performed *T. brucei-*specific PCR. Please see Positive control; Page 20 lines 408-411, Primers distinguish: Page 19 402-406, Detailed information on primer selection is provided in the legend of the S5 Table.

Lines 217-220 mention checking of parasitemia and administering 200ul to mice. What was real and expected parasitemia (concentration)? This should be added to the methodology. In addition, to assess the vector competence, there should be more focus on the infectious dose required to sustain infection in vivo.

**Authors:** Thank you for your comment. The expected parasitemia varied in each stabilate depending on the length of storage and initial parasitemia of the blood before storage in the biobank. However, the parasitemia before inoculation was approximately 1 × 10³ to 1 × 10⁴ trypanosomes/ mL blood. This has been added to the methodology. For the required infectious dose used in the *in vivo* infection experimental assay, the parasitemia was 1 × 10^8^ trypanosome/mL blood. This was achieved after post-inoculation as we had described in the methodology section “Monitoring parasitemia levels in donor mice”. Please see Page 23 line 480

**Reviewer # 1**

**Part III – Minor Issues: Editorial and Data Presentation Modifications**

Authors summary: why are tsetse flies mentioned, if they do not transmit *T. evansi* (in fact *T. evansi* is not able to be transmitted by Glossina flies, but relies on other vectors, including Stomoxys), and *T. vivax* is only mechanically transmitted by Glossinas (and by other vectors as well)

**Author:** Thank you for your question we want to clarify your concern as the follow. We have screened pathogens in *G. pallipides* (Fig.3), trapped *G. pallidipes* in all ecologies as our trap is effective for both, and conducted an *in vivo* transmission experiment of *T. evansi* using *G.pallidipes* utilizing the same *Stomoxys* procedure(please see line 188-191). Our comparative approach mentioned in author summary is supported by our data, aims to capture the attention of both the scientific community, and policymakers, as policy makers tend to give less emphasis to non-tsetse trypanosomiasis vectors

**Reviewer # 1**

African trypanosomes are generally accepted to be T. brucei brucei, T. brucei rhodesiense and T. brucei gambiense. T. evansi and T. vivax are related to them, but they are spread in middle east and America and not generally called “African trypanosomasis”. Generally this should be corrected throughout the manuscript (throughout authors summary, introduction eg. line 72, line 494), better explained and defined, as it is misleading and shows lack of rudimentary knowledge.

**Authors:** Thank you for your comment. This has been corrected as suggested. Please see Page 3 line 38, line 40, line 50, Page 4 line 74, Page 12 249

**Reviewer # 1**

Related to this, the spread of *T. vivax* and *T. evansi* outside the tsetse belt is known and explained by range of vectors, including Stomoxys (lines 77-78). This must be better explained in the work.

**Authors**: Thank you for your comment. We have revised the sentence to better explain this, please see Page 5 lines 81-82

**Reviewer # 1**

Line 85: “capacity to become infected” and ability to “transmit hemopathogens” are two absolutely different things. Especially the infection is not explored in this work and therefore this sentence should be modified

**Authors:** Thank you for your comment, that sentence rewritten. Please see Page 5 lines 88-89

**Reviewer # 1** Lines 116-117 have a typological error in the abbreviation of EDTA

**Authors**: Thank you for pointing this out. This has been addressed accordingly. Please see Page 17 Line 34

**Reviewer # 1**

Lines 127-128 Why were the trapping sites for insects set out of close proximity to villages, if domesticated animals are in focus of the study – would it be better to carry out the sampling near livestock stables, where the transmission of blood-borne pathogens can be more common and Stomoxys flies (stable dwelling flies) would be present?

**Authors:** Thank you for your comment. Except for Kiambu County, all trapping sites were located in wide woodland savannah ecologies, away from villages. The selection of these sites was intentional and part of our study design, as we aimed to capture Stomoxys across diverse ecological settings to assess their potential role as vectors beyond domesticated environments as most wildlife are known reservoirs of these pathogens. This approach allowed us to investigate their diversity, host preference, and pathogen carriers in both livestock-associated and wildlife-rich areas, providing a broader perspective on their epidemiological significance, especially in the wildlife-livestock interface. Please see Page 17 line 354-360

**Reviewer # 1**

Line 129 – methodology for morphological identification of Stomoxys flies – how long did it approximately take from collection to storing the flies at -20°C? Prolonged room-temperature storage could negatively effect the consecutive molecular detection of pathogens.

**Authors**: Thank you for your comment. We pinned a few flies for morphological characterization immediately and the rest were preserved in absolute ethanol that is suitable for DNA-based pathogens and blood meal analysis. Please see Page 18 lines 362-364

**Reviewer # 1**

Line 149: comparing absorbace at 260 and 280 nm is used to assess DNA purity, not concentration, this should be corrected or better explained

**Authors**: Thank you for your observation. This has been revised. Please see Page 19 lines 384-385

**Reviewer # 1**

Line 199 “normal” conditions of mouse housing should be fully described (temperature, night-day cycle, humidity, perhaps even number of animals per cage might be relevant

**Authors:** Thank you for your comment. The conditions under which the mice were kept have been fully described, please see Page 22 lines 450-455

**Reviewer # 1**

Line 221-228 Why was the parasitemia not evaluated during the first three days after infection – was there too low parasitemia expected at early stages of the infection?

**Authors:** Thank you for your comment. Parasitaemia was not evaluated during the first three days post-infection, from our preliminary data we found that parasitemia was too low to be detected using microscopy, i.e. it was below the detection threshold of microscopy which we used to check parasitemia. Please see Page 24 lines 488-490

**Reviewer # 1**

Lines 316-317 describe the distribution of various Stomoxys species – this is interesting data and should be explored further – in figure 3 C, I would welcome a graphical representation of the total number of flies identified, not only percentages.

**Authors**: Thank you for your suggestion. We agree that further exploration of *Stomoxys* species distribution in more ecologies, and seasons is required. We have presented for Kiambu county the flies density for the whole year per month, but for other sites due to distance from *icipe,* we did not do such an experiment. However, we presented flies density from all counties as supplementary data (S2 table.).

**Reviewer # 1**

Line 411 describes that “Stomoxys feeds about 9.98 ± (5.5) mL blood”. I hope that this is a typological error and should be microliters and not mililiters. In addition, this statement should be supported by citation.

**Authors**: Thank you for identifying this error. You are right this should be µL rather than mL.

We have corrected this typing error. Concerning the 9.9 µL of blood ingested by Stomoxys is an original data, our initial submission lacked clarity since we provided only the numerical value. We appreciate your inquiry; we have now detailed the methodology and included the raw data, in (S4 table), please see Page 9 lines 168-169

**Reviewer # 1**

Line 424 mentions clinical symptoms in mice, these should be described.

**Authors:** Thank you for your comment. The observed symptoms have been added, please see Page 9 line 181-182

**Reviewer # 1**

Figure 6 legend should be described in more detail

**Authors**: Thank you for the comment. The legend has been described more. Please Fig 6 Legend

**Reviewer # 1**

Line 483-484 Mentions strained animals, what does this mean, should it be “starved” instead? Also blood meal is given in mg instead of ml, in contrast to previous mention in the text. This should be also supported by citation

**Authors:** Thank you for pointing out this error. We have rephrased the sentence for a better understanding. It should be “restrained”. We have also changed the unit of measurement from mg to µL, and we described the detail for your previous inquiry. Please see Page 12 lines 237-238

**Reviewer # 1**

Lines 514-516 mention lack of detection of Ehrlichia and Rickettsia – I find this confusing. Were the PCR primers used to detect the pathogens in hosts, but failed to detect them in the pathogen?

**Authors:** Thank you for your concern.

We successfully detected *Ehrlichia* spp. in livestock but not in *Stomoxys* and *G. pallidieps*, we failed to get quality sequence for *Rickettsia* thus we omitted the Rickettsia result. The absence of the pathogens in flies is not because of the primers, our PCR primers are specific and performing as it detected the pathogens and resulted in quality sequence however, we got negative results when screening both vectors for the pathogens, showing they did not harbour the pathogens for unknown reason. Please see Page 13 lines 266-267.

**Reviewer # 1**

Lines 546-547 sentence is missing a full stop.

**Authors:** Thanks, full stop added. Please see Page 28 lines 576-577.

**Reviewer # 1**

Lines 556-557 in conclusion state that “Stomoxys flies may play a significant role in the spread and maintenance of *T. evansi* and *T. vivax* in the wide geographic regions of the world.” This is a known fact

**Authors:** We have explained to your previous question in the author summary which is highly related we have supported the claim with strong data, on blood feeding, pathogens and distribution and we showed less than a minute is enough to transmit pathogens showing how stomoxys are efficient in transmitting multiple pathogens. Please see Page 15 lines 308- 311

**Reviewer # 1**

Linguistic concerns: the whole text should be re-checked for grammatical coherence and mistakes. Some minor changes include line 198: live body weight should be “body weight” only, mouse age should be precise range etc. Line 412 states “[needs] minutes to fully engorged” – missing “be”. Similar mistakes are present throughout the text and should be found and corrected by the authors.

**Authors:** Thank you for pointing out the linguistic concerns. They have been addressed throughout our manuscript.

**Reviewer # 2**

**Part I - Summary**

Reviewer #2: This is a very interesting paper with a novel approach to pathogen transmission in stable flies. The science is good, but the writing is wordy and the paper probably could be trimmed down a bit. The English is good, but sometimes it appears that 2 different people wrote various sections. Most of my comments are merely about housekeeping. for example, when a product is first mentioned, with accompanying source information, the source info does not need to be repeated each timed the product is subsequently mentioned. I made changes in the text for clarity and brevity. There is plenty of information in the text and clarity is important. There are no Key Words. Are they not required? The title is not very specific. Although the results indicated transmission of several hemopathogens, the most specific work was done with 2 species of Typanosomes. A title that read "......competent vectors of Trypanosoma vivax, T. evansi, and multiple livestock hemopathogens" would have searchable hemopathogen names. The stable fly host list of domestic and feral African animals, including humans, is a good addition to the literature. Stomoxys species grouping studies are valuable for future work. Figures are good but hope the published font size will be larger. See other suggested changes in the text

**Authors:** Thank you for your positive and critical comments about work, that ahs improved the clarity of the manuscript.

•	We have revised the manuscript to improve clarity and conciseness.

•	Redundant product source information has been removed after its first mention (also as seen in the track changes).

•	For the Key Words they are not a requirement of the PLOS Pathogens journal.

•	Regarding the title, thanks for your suggestion and we agree that more specificity in it will enhance searchability, and we have revised it to: “Stomoxys flies (Diptera, Muscidae) are competent vectors of *Trypanosoma evansi*, *Trypanosoma vivax*, and other livestock hemopathogens”

•	We have also edited the figures to .tif format which will be readable in the final publication.

**Reviewer # 2**

**Part II – Major Issues: Key Experiments Required for Acceptance**

Reviewer #2: There are more than enough experiments discussed in the text to support the results.

**Authors:** Thank you for your feedback. We appreciate your positive evaluation of the experimental work presented in the manuscript.

**Part III – Minor Issues: Editorial and Data Presentation Modifications**

Reviewer #2: Minor issues refer mainly to writing brevity and clarity. Data are clear as is. Writing changes can be found throughout.

**Authors:** Thank you for your comment. We also appreciate your track changes in the manuscript. We have addressed them accordingly. We have improved brevity and clarity throughout the manuscript. Also, we have worked on the grammatical and syntax errors.
